# Cool Temperatures Reduce Antifungal Activity of Symbiotic Bacteria of Threatened Amphibians – Implications for Disease Management and Patterns of Decline

**DOI:** 10.1371/journal.pone.0100378

**Published:** 2014-06-18

**Authors:** Joshua H. Daskin, Sara C. Bell, Lin Schwarzkopf, Ross A. Alford

**Affiliations:** School of Marine and Tropical Biology, James Cook University, Townsville, Queensland, Australia; Clemson University, United States of America

## Abstract

Chytridiomycosis, caused by the fungus *Batrachochytrium dendrobatidis* (*Bd*), is a widespread disease of amphibians responsible for population declines and extinctions. Some bacteria from amphibians’ skins produce antimicrobial substances active against *Bd*. Supplementing populations of these cutaneous antifungal bacteria might help manage chytridiomycosis in wild amphibians. However, the activity of protective bacteria may depend upon environmental conditions. Biocontrol of *Bd* in nature thus requires knowledge of how environmental conditions affect their anti-*Bd* activity. For example, *Bd*-driven amphibian declines have often occurred at temperatures below *Bd*’s optimum range. It is possible these declines occurred due to reduced anti-*Bd* activity of bacterial symbionts at cool temperatures. Better understanding of the effects of temperature on chytridiomycosis development could also improve risk evaluation for amphibian populations yet to encounter *Bd*. We characterized, at a range of temperatures approximating natural seasonal variation, the anti-*Bd* activity of bacterial symbionts from the skins of three species of rainforest tree frogs (*Litoria nannotis, Litoria rheocola,* and *Litoria serrata*). All three species declined during chytridiomycosis outbreaks in the late 1980s and early 1990s and have subsequently recovered to differing extents. We collected anti-*Bd* bacterial symbionts from frogs and cultured the bacteria at constant temperatures from 8°C to 33°C. Using a spectrophotometric assay, we monitored *Bd* growth in cell-free supernatants (CFSs) from each temperature treatment. CFSs from 11 of 24 bacteria showed reduced anti-*Bd* activity *in vitro* when they were produced at cool temperatures similar to those encountered by the host species during population declines. Reduced anti-*Bd* activity of metabolites produced at low temperatures may, therefore, partially explain the association between *Bd*-driven declines and cool temperatures. We show that to avoid inconsistent antifungal activity, bacteria evaluated for use in chytridiomycosis biocontrol should be tested over a range of environmental temperatures spanning those likely to be encountered in the field.

## Introduction

Emerging wildlife diseases can cause species declines and extinction [1], and disease emergence and pathogenicity may depend on environmental context [2]. Therefore, patterns of decline and disease management strategies may be understood by examining the effects of environmental context on disease emergence. One way environmental context might affect disease dynamics is by altering species interactions in the complex assemblage of microbiota inhabiting wildlife [3]. Chytridiomycosis, a disease caused by the fungal pathogen *Batrachochytrium dendrobatidis* (*Bd*) and responsible for rapid and extensive population declines in over 200 amphibian species since the late 1970s [4], [5], serves as a model system for understanding other wildlife diseases [6]. Twenty years of research on *Bd* and its interaction with amphibians and their environment can provide insight for anyone interested in the effects of diseases on the conservation of wildlife [5], [7].

Currently, no effective treatments or preventative actions are available to manage chytridiomycosis in wild populations of threatened amphibians [8]. However, bioaugmentation, by supplementing populations of anti-*Bd* bacteria, has proved effective in laboratory trials and may be a viable option for disease management if it also provides increased protection in nature [9]–[11]. Previous experimental work on anti-*Bd* bacteria was conducted under constant laboratory conditions [11], [12]. However, naturally-occurring symbiotic bacteria, including antibiotic producers, vary in abundance and physiological activity with environmental context [13]–[15]. This variation can affect the success of biocontrol programs [16]–[18], and can have strong evolutionary and ecological impacts [3]. Bacteria chosen for probiotic use based on high levels of anti-*Bd* activity under constant conditions in the laboratory could have lower levels of anti-*Bd* activity in the more variable conditions occurring in nature. If this occurs, then choosing bacteria for use in the management of chytridiomycosis, will require knowledge of how candidate bacteria are affected by a range of environmental conditions [9].

Environmentally induced variation in the protection afforded to amphibian hosts by bacterial symbionts might also partially explain patterns of past chytridiomycosis-driven declines. In the tropics, where the impacts of chytridiomycosis have been most severe, higher elevations and cooler seasons have been associated with higher prevalences of infection, more intense infections, and more frequent declines [19]–[24]. *Bd*’s relatively cool thermal optimum (17–25°C) [25] may partially explain this pattern [21], but many declines have occurred at temperatures well below this window, where one might expect chytridiomycosis to be less severe [26].

There are a number of reasons why declines might occur at very low temperatures. In natural environments, *Bd* may respond to low temperatures by increasing fecundity [27], and amphibian hosts’ immune defenses may be weaker [28]. Another possible contributor to the high incidence of *Bd*-driven declines at temperatures below *Bd*’s *in vitro* thermal optimum is that symbiotic cutaneous bacteria, which would otherwise reduce the severity of chytridiomycosis, may have reduced activity or population density at cooler temperatures. At present, there is no published information on how the composition or antifungal activity of assemblages of anti-*Bd* bacteria responds to changes in environmental context.

To examine the effect of temperature on the production of anti-*Bd* metabolites by bacteria, we sampled bacterial symbionts from the Australian hylid frogs *Litoria serrata* and *Litoria nannotis* in 2010. We also examined bacteria isolated by Bell [29] and Bell et al. [30] from *Litoria rheocola, L. serrata*, and *L. nannotis* in 2009. All three species experienced population declines following chytridiomycosis outbreaks, although some populations have since recovered or recolonized [23], [31]. We identified the bacteria and characterized their antifungal activity across a range of the temperatures experienced by their amphibian hosts.

## Materials and Methods

### Ethics Statement

All procedures involving animals received clearance from the James Cook University Animal Ethics Review Committee (approval number A1316) and all field sampling was permitted by the Queensland Department of Environment and Resource Management (permit WITK05922209).

### Collection and Isolation of Bacteria

To screen for anti-*Bd* activity, we collected bacteria from twelve frogs (six *L. serrata*, six *L. nannotis*) caught at Windin Creek in Wooroonooran National Park, Queensland, Australia (∼750 m a.s.l., S 17°21′57″ E 145°42′54″) in February 2010. We rinsed each frog with a stream of sterile distilled water to remove non-resident bacteria [32], [33], then swabbed its dorsal and ventral surfaces and legs twice, using a sterile rayon swab (MW112, MWandE, Bath UK). We streaked each frog’s swab onto a low nutrient agar plate (R2A, Becton, Dickinson and Company, New Jersey, US). We used new gloves and plastic bags to catch, handle, and hold each animal to prevent disease transmission or contamination of samples. We released frogs at their point of capture immediately after swabbing. After returning our samples to the laboratory, we isolated each morphologically-distinct bacterium into axenic (pure) culture using standard microbiological techniques, and checked Gram stains of each isolate using light microscopy to ensure purity [34]. Axenic isolates were stored on ceramic Microbank microbeads (Microbank, Pro-Lab Diagnostics U.K.). The beads are stored dry at −80°C. When a live culture is required, a bead can be dropped into liquid media or streaked across solid agar.

### Identification of Anti-Bd Bacteria

To determine whether bacterial isolates inhibited growth of *Bd* *in vitro*, we performed challenge assays using a method developed by Bell et al. [30] with slight modifications described here. We inoculated bacteria from Microbank microbeads into one mL TGhL broth (eight g tryptone, one g gelatine, two g lactose per liter of water) in 24-well plates (Costar 3524, Corning, New York, US) and incubated them for 48-hours at 23°C. We then centrifuged each liquid culture for five minutes at 7500×g, and filtered the supernatant through a 0.22 µm syringe filter (Millex GV, Millipore, Massachusetts, US). This left a cell-free supernatant (CFS) containing bacterial metabolites.

In 96-well assay plates (Costar 3595, Corning, New York, US), we inoculated 1.0×10^5^ live *Bd* zoospores (isolate Gibbo River, L. Les, 06-LB-1 isolated by L. Berger in 2006 from a *Litoria lesueri* and passaged weekly) suspended in 50 µL of fresh TGhL into each of five replicates containing 50 µL of each CFS. We included replicates of positive and negative controls consisting of 1.0×10^5^ live and heat-killed *Bd* zoospores, respectively, suspended in 100 µL TGhL. We incubated the plates at 23°C and monitored progress of *Bd* growth in each well with daily spectrophotometric readings at 492 nm [35]. We continued monitoring until maximum growth was observed in the positive control and in the majority of wells containing CFS (four days). This is the most conservative point in the assay at which to determine anti-*Bd* activity; i.e., this is the point at which the ratio of *Bd* growth in CFSs to *Bd* growth in the positive control is maximized. We examined 96-well plates using light microscopy and excluded from our analyses any replicates that had become contaminated.

We transformed mean optical densities at 492 nm (OD_492_) for each isolate-temperature combination on each day, to correct for initial coloration of CFSs (by subtracting the mean initial OD_492_) and background absorbance of inoculated zoospores on that day (by subtracting the mean negative control OD_492_) [30]. Using the corrected value on the maximum growth day for both the positive control and for each CFS by temperature combination, we standardized isolate-specific values against the similarly corrected value for the positive control. This produced a measure of *Bd* growth in each CFS by temperature combination as a proportion of that in the positive control. Finally, we multiplied the corrected and standardized values by 100 percent. This returned the following values: 100% for the positive control; 0 for total inhibition of *Bd* growth; >0 but <100% for partial inhibition, and >100% for CFSs in which *Bd* grew better than in the positive control. Thus, the values are the percent *Bd* growth relative to that in the positive control. Following Bell et al. [30] we considered CFSs in which *Bd* growth was reduced on average at least 63.5% below that in the positive control to be strongly inhibitory. This value is a conservative correction for the maximum observed effects of nutrient depletion in the media carried into challenge assay wells along with inoculated CFSs. When *Bd* is inhibited more than 63.5%, it is assumed to be true CFS-driven inhibition, and not due to nutrient depletion [30].

We extracted DNA from each isolate that strongly inhibited *Bd*, first by three freeze-thaw cycles of 10 minutes each at −80°C and 70°C, and then, if freeze-thaw cycles alone did not yield sufficient DNA for successful PCR, using a Qiagen (Hilden, Germany) DNeasy blood and tissue kit with pretreatment for Gram-negative bacteria, as per the manufacturer’s protocol. DNA was amplified using universal bacterial 8F and 1492R primers [36], and sequenced by Macrogen, Inc. (Seoul, South Korea). We aligned forward and reverse sequences in Geneious [37] and matched to sequences in the NCBI GenBank database (http://ncbi.nlm.nih.gov) to identify bacteria. We have submitted genetic sequence data to GenBank.

### Experimental Challenge Assays

To test for temperature-induced changes in bacterial anti-*Bd* activity we performed additional challenge assays and quantitative analysis using bacteria identified in the initial screening assay as strongly inhibitory. In addition to the bacteria isolated from frogs sampled in February 2010, we included strongly inhibitory bacteria isolated from *Litoria nannotis*, *L. serrata*, and *L. rheocola* at Windin Creek in the Austral winter of 2009 and tested using the methods described above [29]. In total, we tested 24 isolates in the experimental challenge assays, all of which inhibited *Bd* growth by more than the 63.5% threshold in initial challenge assays (Table 1).

**Table 1:** Bacteria identified as strongly inhibitory of *Batrachochytrium dendrobatidis* and used in the experimental challenge assay.

Taxonomic name	Frog species‡, Year	GenBank Accession ID, % Matching*	GenBank Accession Number
Actinobacteria			
*Microbacterium* sp. HY14(2010)	LN, 2010	HM579805, 99.5	KJ191412
Bacilli			
*Bacillus thuringiensis* isolate CCM15B	LN, 2010	FN433029, 99.8	KJ191418
β-Proteobacteria			
Uncultured *Silvomonas* sp. clone ntu63	LS, 2010	EU159476, 98.5	KJ191396
Bacterium H2	LR, 2009	AY345552, 99.1	KJ191380
***Iodobacter*** ** sp. CdM7**		**FJ872386, 98.8**	
Flavobacteria			
*Chryseobacterium* sp. CH33	LN, 2009	GU353129, 99.1	KJ191375
Uncultured bacterium clone nbw1150f04c1	LS, 2010	GQ082309, 99.1	KJ191421
***Chryseobacterium hispanicum*** ** type strain VP48**		**AM159183, 98.7**	
γ-Proteobacteria			
*Hafnia alvei*	LN, 2009	AB519795, 99.9	KJ191378
*Pseudomonas fluorescens* strain 1408	LS, 2009	GU726880, 99.9	KJ191384
*Pseudomonas fluorescens* strain d3_16s	LS, 2010	HQ166099, 99.7	KJ191426
*Pseudomonas fluorescens* strain KU-7	LR, 2009	AB266613, 98.9	KJ191386
*Pseudomonas koreensis* strain Ps 9–14	LR, 2009	NR025228, 99.9	KJ191376
*Pseudomonas koreensis* strain SSG10	LN(3), 2010	HM367598, 99.8; 99.8; 99.7	KJ191409
*Pseudomonas koreensis* strain SSG5	LN, 2010	HM367599, 99.9	KJ191405
*Pseudomonas mosselii* strain WAB1873	LN, 2010	AM184215, 99.7	KJ191414
*Pseudomonas mosselii* strain R10	LN, 2010	DQ073452, 99.6	KJ191411
*Pseudomonas putida* strain PASS3-tpnb	LR, 2009	EU043325, 99.7	
*Pseudomonas* sp. SBR3-slima	LN, 2010	EU043328, 99.3	KJ191420
*Pseumonas tolaasii* strain NCPPB 2325	LR, 2009	AF320990, 100	
*Serratia marcescens* strain C1	LS(2), 2010	GU220796, 99.9; 99.7	KJ191397
*Stenotrophomonas maltophilia* strain 6B2-1	LR, 2009	AB306288, 99.9	KJ191382
*Stenotrophomonas maltophilia* strain YLZZ-2	LS, 2009	EU022689, 99.6	KJ191387
*Stenotrophomonas* sp. 7-3	LR, 2009	EU054384, 99.6	KJ191381
Uncultured bacterium clone nbw969a06c1	LN, 2010	GQ043359, 98.4	KJ191393
***Xanthomonas*** ** sp. CC-AFH5**		**DQ490979, 98.1%**	
Uncultured bacterium clone P7D82-747	LN, 2010	EF509545, 99.6	KJ191417
***Stenotrophomonas*** ** sp. TSG4**		**HM135101, 99.6%**	

Where the closest GenBank match was an unnamed bacterium, the closest named match is included immediately below (smaller, bold text) to give the best possible sense of phylogeny.

*Where more than one inhibitory isolate most closely matched the same OTU, the percent matching is listed for both. We used the isolate with the first listed percent matching for the experimental challenge assay.

‡ LN = *Litoria nannotis*, LR = *Litoria rheocola*, LS = *Litoria serrata*; (number of individuals from which the bacteria was isolated, if >1).

We inoculated each bacterium from a Microbank microbead into a 25 cm^2^ flask (TPP, Trasadingen, Switzerland) containing 10 mL TGhL broth and incubated it at 23°C for at least 48 hours until growth was observed. We then added 500 µL of each bacterial inoculum to one mL TGhL in each of six, 24-well plates. One plate was placed in each of six incubators set at 8, 13, 18, 23, 28, and 33°C, respectively. We chose temperatures to approximate the range of conditions experienced by *Litoria* spp. in the Australian Wet Tropics [38].

We grew bacterial cultures for two to five days, based on the time to maximal absorbance at 492 nm (a surrogate for maximal bacterial concentration) and used these cultures to produce the CFSs included in the next round of challenge assays. Growing cultures to maximum absorbance minimized any differences among treatments that might have arisen if metabolite production was triggered by quorum sensing, a mechanism of detection and response in bacterial colonies based on population density [39]. When OD_492_ stopped increasing (qualitatively determined from the plateauing of absorbance values) we produced bacterial CFSs as described for the initial screening assay. We harvested each individual bacterium as it reached maximum growth; i.e, we did not wait for absorbance of all bacteria in a plate to plateau before harvesting. This method does not account for possible differences in the maximum achievable concentration of any given bacterium at different temperatures. CFSs were held at −20°C until use in the challenge assays. We completed challenge assays at 23°C with five replicates inoculated from each growth treatment temperature for each bacterium’s CFS, and with 2.9×10^4^ zoospores initially inoculated in each 100 µL assay well. *Bd* was therefore not cultured at a range of temperatures; only the bacteria used to produce CFSs were. This allowed us to evaluate the effects of temperature on the bacteria independent of the well-known effects of temperature on the growth of *Bd* [25]. We removed from the analysis any replicates that appeared to be contaminated when observed by light microscopy. As in the initial assays, the challenge assays continued until maximum growth was achieved in the positive controls (here six days).

### Statistical Analysis

Corrected and standardized absorbance data were averaged across replicates of each CFS-temperature combination. The resulting averages were not normally distributed and could not be normalized by standard transformations. Therefore, we used a Kruskal-Wallis test to examine the effects of temperature on the anti-*Bd* activity of CFSs from tested bacteria. We performed analyses in S-PLUS (version 8.0, Insightful Corporation, Seattle) and R 3.0 (R core team, 2013).

## Results

### Identification of Anti-Bd Bacteria

**Figure 1:**
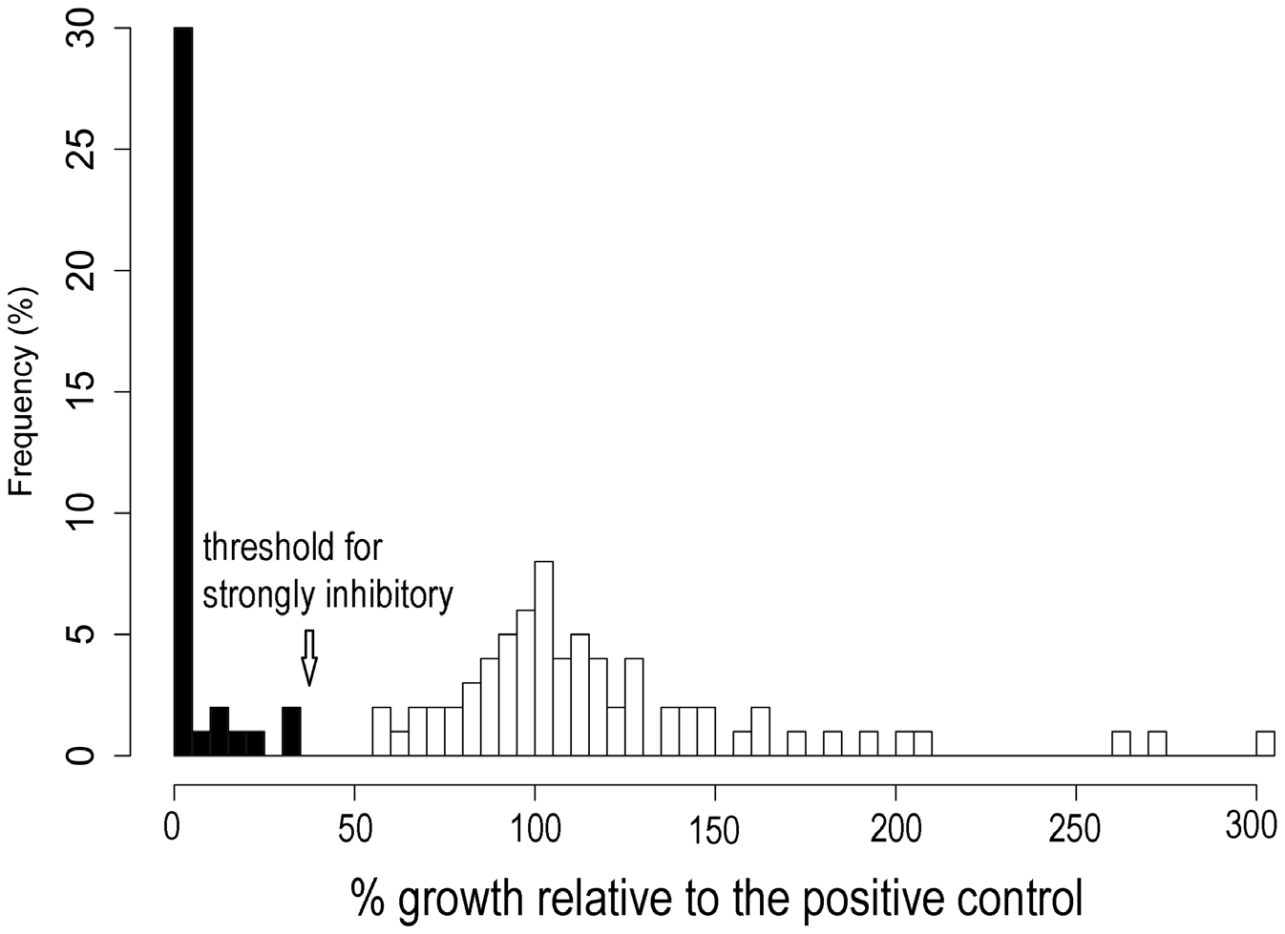
Frequency (%) of the bacterial isolates which produced cell-free supernatants (CFS) showing a change in *Bd* growth relative to the positive control (*Bd* alone; 100%) for 110 isolates screened in the initial challenge assay. A bimodal pattern of activity was observed. The arrow indicates the cutoff for considering a CFS strongly inhibitory (≥63.5% inhibition relative to the positive control, i.e., ≤36.5% the growth of the positive control). Thus, black bars represent strongly inhibitory CFSs and the highest bar at the far left represents those isolates producing cell-free supernatants showing 100% inhibition.

**Figure 2:**
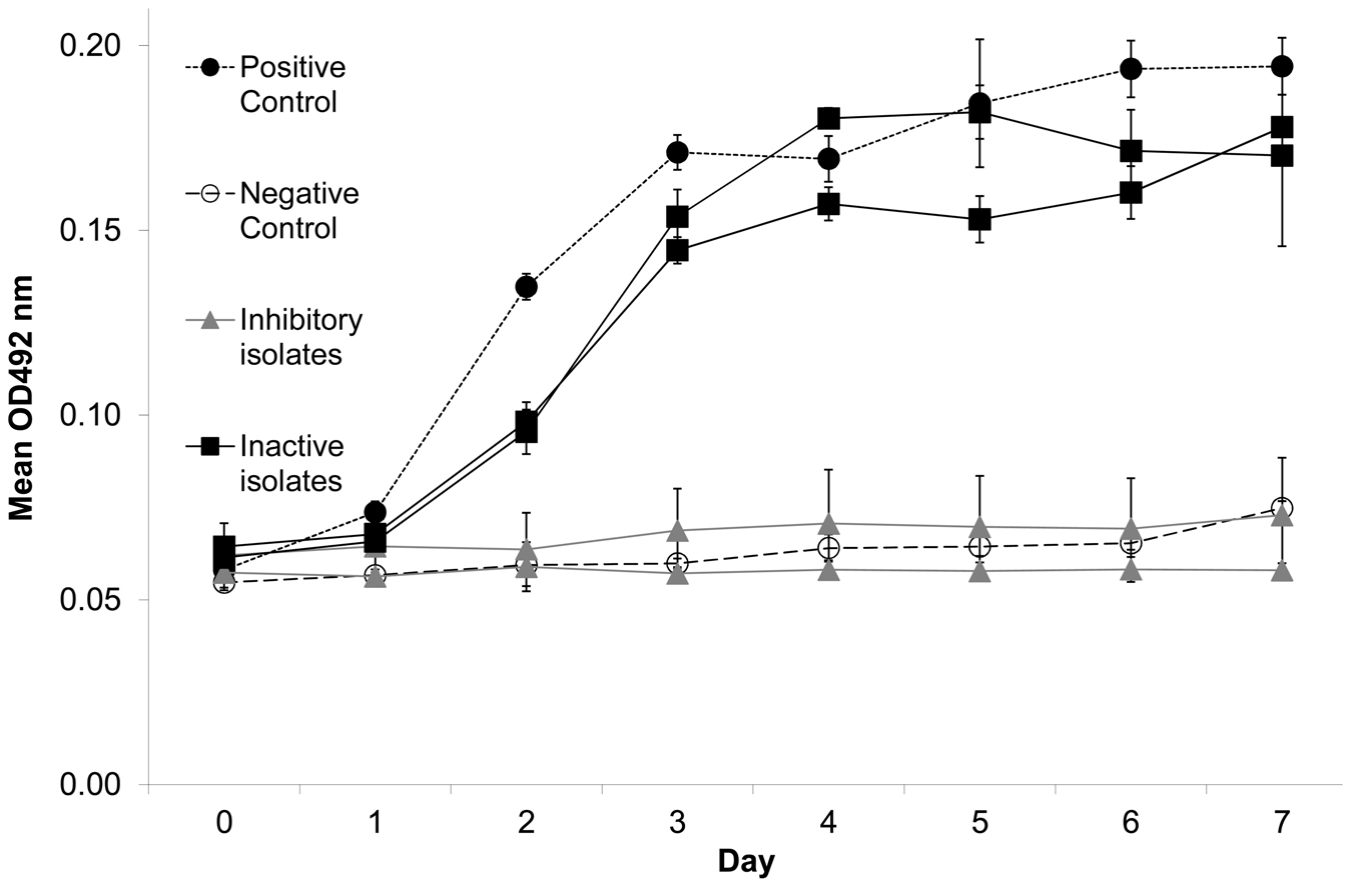
Example growth curves ±SD of the positive control, the negative control, two inactive, and two strongly inhibitory cell-free supernatants from an initial screening assay. Higher optical density at 492(y-axis, OD_492_) indicates greater *Bd* growth. Day 0 OD_492_ varies with original cell-free supernatant color, which is corrected for in calculations (see Methods).

**Figure 3:**
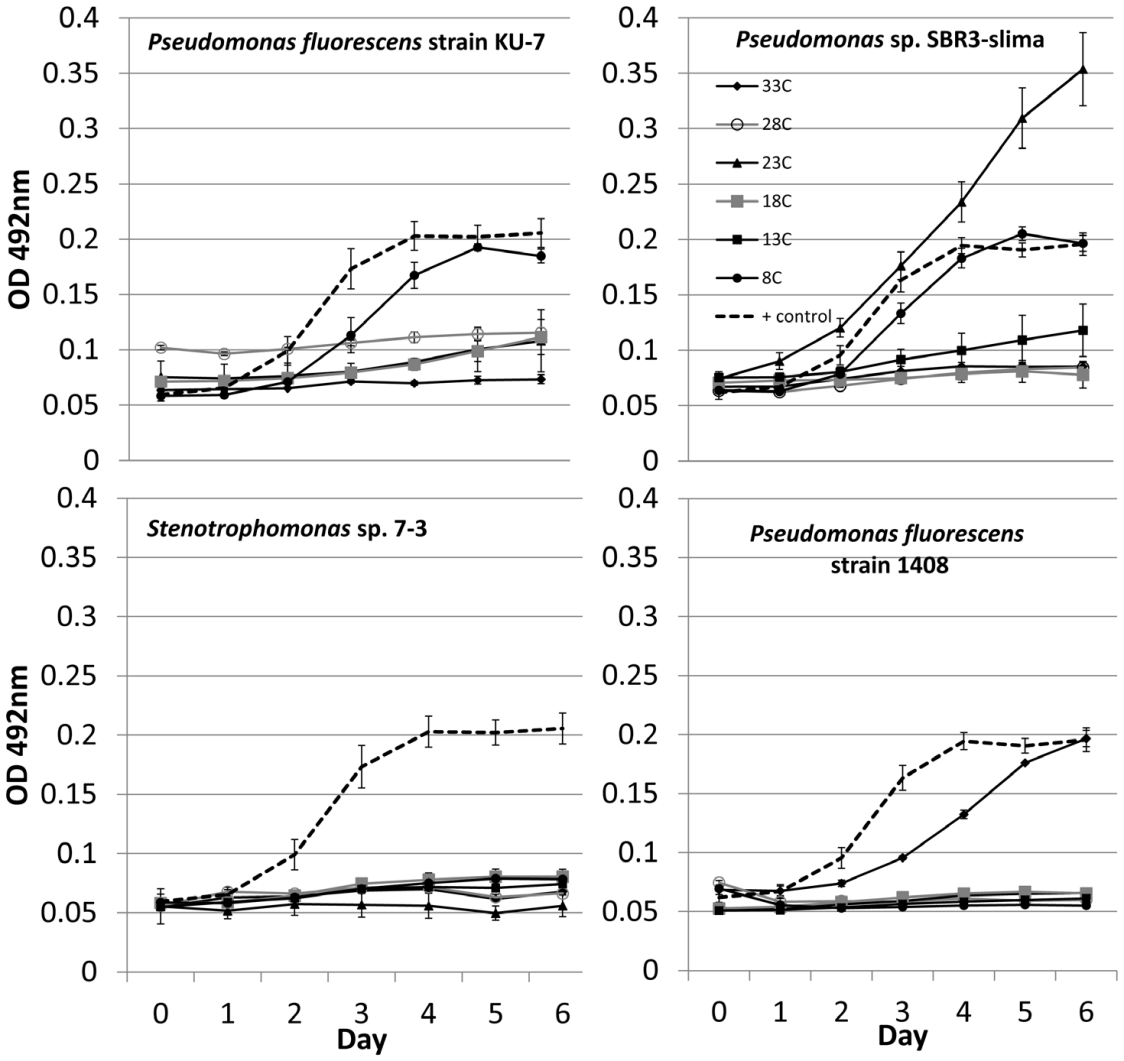
Growth curves (uncorrected optical density at 492 nm, OD 492 nm) vs. day of the experimental challenge assay for *Bd* growth in cell-free supernatants from four bacteria chosen as examples to show the range of responses observed following the temperature treatments applied during cell-free supernatant production. Values are means ±SD. Where not visible, SD bars are smaller than the plotted symbols. All bacterium-temperature combinations have N = 5 replicates in the challenge assay, except *P. fluorescens* with N = 4 at 23°C and 18°C, N = 3 at 33°C and no data at 13°C due to contamination. Cell-free supernatants were produced once at each temperature.

Four of 720 replicates (0.6%) were removed from the initial challenge assay due to contamination. *Bd* growth expressed relative to the positive control was not normally distributed among CFSs in the initial screening assay (Figure 1). Thirty-seven percent of CFSs tested at this stage inhibited *Bd* growth strongly. *Bd* grew poorly or not at all when exposed to these CFSs (Figure 2). We also observed a small number of CFSs (e.g., *Pseudomonas* sp. SBR3-slima, Figure 3) that apparently enhanced *Bd* growth, a phenomenon also reported by Bell et al. [30].

After removing duplicate isolates that came from the same individual frog and matched identical GenBank entries, 16 distinct, strongly inhibitory bacterial isolates remained from samples collected in 2010. These 16 matched the 13 operational taxonomic units (OTUs) listed for 2010 in Table 1; one OTU was found on two frogs, and one was found on three. Pseudomonads constituted six of the 13 OTUs, making them the most common group among inhibitory bacteria. The strongly inhibitory isolates collected in 2009 and included in experimental assays are also listed in Table 1.

### Experimental Challenge Assay

The experimental challenge assay continued for six days, at which point *Bd* in the positive control and in 136 of 157 CFSs had reached its maximum growth. All the 21 remaining CFSs had *Bd* growth and absorbances already well above the point at which they would have been considered inhibitory. Forty-one of 785 replicates (5.2%) became contaminated and were subsequently removed from analysis of the initial challenge assay. Bacterial responses to temperature included all of increased, decreased, and non-directional changes to CFS anti-*Bd* activity (Figure 3).

**Figure 4:**
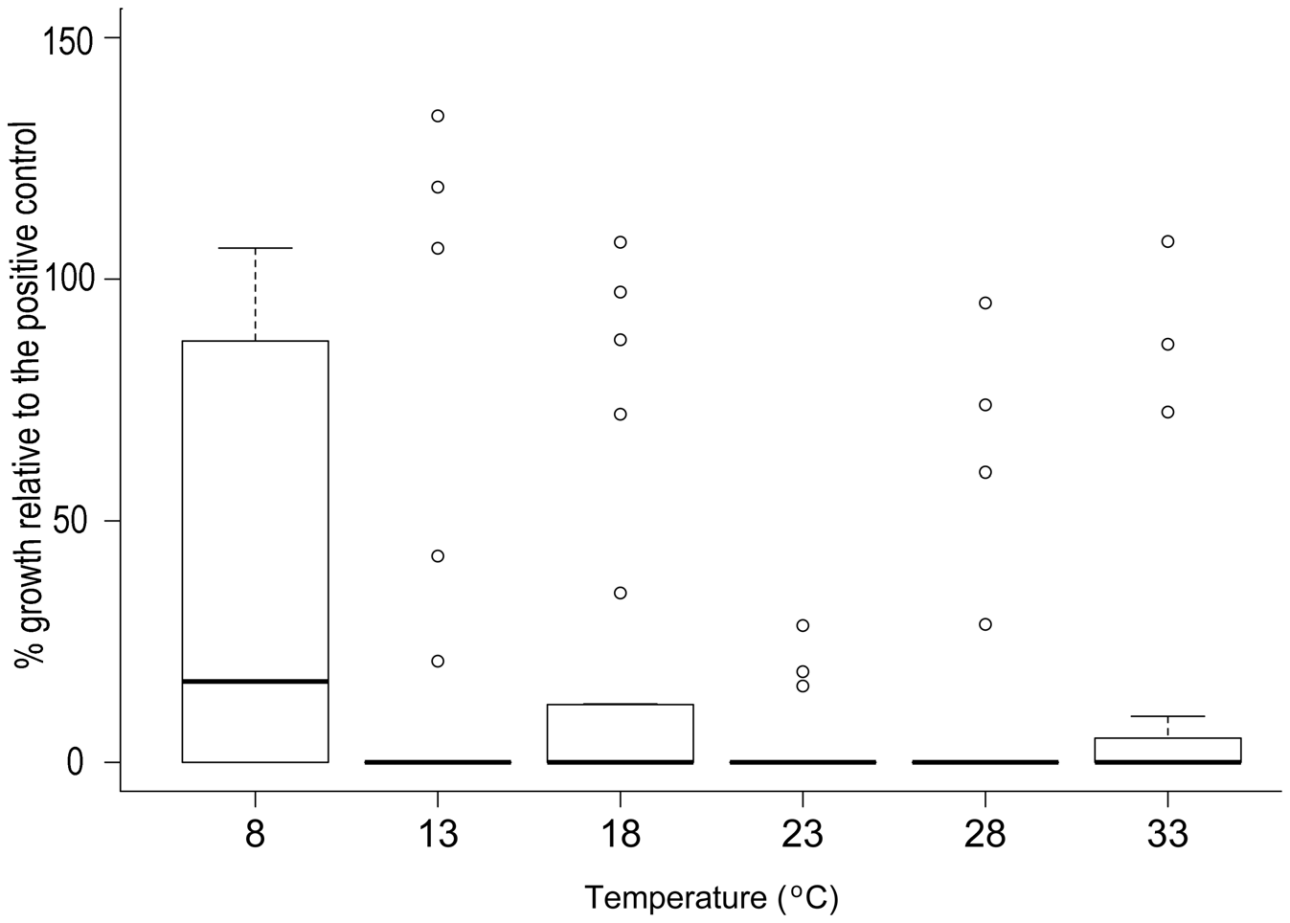
Percent *Bd* growth relative to the positive control (*Bd*-alone; 100%) in bacterial cell-free supernatants from 24 bacteria identified in initial screening at 23°C as strongly inhibitory to *Bd* and grown in each of six temperature conditions. A value of 0 indicates complete inhibition of *Bd* growth, and a value of 100% indicates growth equivalent to that of the positive control (for details see above and [30] ). Boxes show the interquartile range (IQR) and the median. Brackets are the most extreme values to 1.5X the IQR, and individual points are those beyond this span. Where boxes are not visible, IQR was near zero.

The temperature at which bacteria were cultured significantly affected the antifungal activity of their CFSs (Kruskal-Wallis test, χ^2^ = 15.35, df = 5, p<0.01). Antifungal activity tended to be lowest in CFSs produced at 8°C (Figure 4). Using the cutoff from the initial screening assay of 63.5% or greater inhibition of *Bd* growth relative to the positive control, 46% of tested CFSs had no strong antifungal activity when produced at 8°C, whereas no more than 28% produced at any of the warmer temperatures lost strong antifungal activity.

Bell et al. [30] observed various fungicidal (leading to the breakdown of *Bd* cells) and fungistatic (arresting *Bd*’s life cycle) effects of CFSs on *Bd*. We observed similar effects in the cultures we monitored visually by light microscopy. For example, in the CFSs of *Pseudomonas fluorescens* strain 1408 grown at 8°C, the zoospores of *Bd* appeared dead and deformed, whereas in the CFSs of *Chryseobacterium* sp. CH33 grown at 8°C, zoospores developed into sporangia without producing viable zoospores.

## Discussion

### Variation in Antifungal Activity

The metabolites of many of the defensive bacterial symbionts of amphibians we tested had reduced antifungal activity when produced at 8°C. Such reductions in anti-*Bd* activity were likely caused by changes in the quantity, identity, or both of bacterially-produced substances, and could have contributed to chytridiomycosis-driven declines that occurred in high elevation populations of the three *Litoria* species sampled here [23], [31], [40]. Winter air temperatures in the high elevation habitats of these species can often be lower than 8°C [26], [41]. Therefore, frogs may experience decreased bacterial protection from *Bd* in winter, which in Australia and elsewhere is when chytridiomycosis causes greater morbidity and mortality [19], [23], [42]. While *Bd* physiology may also be altered under variable temperature regimes and it will be important to test bacteria and *Bd* exposed together to a range of temperatures, our design and the *in vitro* assay we used did not allow for testing such effects. Nonetheless, our experiment is an incremental step towards understanding context-dependency in amphibian-*Bd*-bacteria interactions.

Anti-*Bd* bacteria occur on a wide geographic and phylogenetic range of amphibians [12], [43], [44], and the pattern that chytridiomycosis is more virulent at cool temperatures is also widespread [19]–[21], [23]. Many bacteria can alter their rates of antibiotic production in response to environmental temperatures [17], [45], [46]. Like the *Litoria* spp. we sampled, other amphibians may be more vulnerable to chytridiomycosis at cool temperatures if their bacterial protection from *Bd* is reduced. Larger scale, longitudinal studies characterizing the diversity and abundance of amphibian bacterial symbionts and their metabolites, *Bd* infection loads, and chytridiomycosis severity across environmental gradients and among seasons, are required to test this hypothesis.

Although the general trend was towards decreased anti-*Bd* activity when CFSs were produced at cooler temperatures, some bacteria did not show unidirectional responses to temperature (e.g., *Pseudomonas* sp. SBR3-slima, Figure 3); they produced metabolites without anti-*Bd* activity at the lowest temperature and at moderate temperatures. These complex temperature responses may have been caused by production of different antibiotics at different temperatures. Many bacteria produce more than one antibiotic [45], and some regulate their production through multiple genes [47] that could possess different temperature thresholds.

We selected the bacteria we tested in the experimental challenge assay because their supernatants were inhibitory when produced at 23°C in the initial screening assay. It is possible that a different subset of the entire sampled bacterial community could have been classed as inhibitory if the CFSs for the initial screening assay had been produced at different temperatures. One of our aims, however, was to document context-dependency in bioaugmentation candidates identified as inhibitory in ‘standard’ challenge assays conducted at 23°C [12], [30]. Conducting this sort of laboratory assay under ecologically-relevant conditions has been identified as a necessary step towards selecting effective bioaugmentation strategies [9]. However, it is entirely possible that some bacteria that live on the skin of the *Litoria* species we sampled are effectively antifungal at low temperature and were not selected because they are not inhibitory at 23°C. Equally, the possibility of density-dependent responses of *Bd* to CFSs, if for example zoospores can degrade some bacterial products in the CFSs, means that had we conducted the initial challenge assay using more or fewer zoospores, different bacteria may have been identified as inhibitory and subsequently included in the experimental challenge assay. We used slightly different concentrations of *Bd* zoospores in the initial and experimental challenge assays due to temporal variation in the productivity of laboratory *Bd* cultures. However, all CFSs tested in the experimental challenge assay were inoculated with the same concentration of *Bd* zoospores and therefore any possible density-dependent responses of *Bd* to CFSs could not have affected the temperature-driven effects on CFS anti-*Bd* activity reported here.

To produce CFSs for the experimental challenge assay, we grew bacterial cultures to maximum absorbance in each temperature treatment. Because some bacteria may have been dying and their cells breaking apart at this stage, it is possible that these by-products, in addition to anti-fungal metabolites produced by live bacteria, could be partially responsible for observed anti-*Bd* activity. If bacteria died and broke apart to a greater extent in the higher temperature treatments, it could explain some of the reduced anti-*Bd* activity at 8°C. Further experiments employing chemical methods will be needed to definitively identify and evaluate the products responsible for differential anti-Bd activity of CFSs.

Our present study is the latest to find a substantial number of *Pseudomonas* species, a well-known group of antibiotic producers [45], [48], among the antifungal cutaneous microbiota of amphibians [12], [30], [40], [49]. Walke et al. [44] offered several ecological and physiological reasons for the prevalence of Pseudomonads among the defensive microbiota of amphibians. Our results show that the anti-*Bd* activity of at least some Pseudomonads depends on environmental context (e.g., *Pseudomonas* sp. SBR3-slima, Figure 3). Only the most robustly antifungal isolates should be used in bioaugmentation, regardless of how common they may be.

### Management Implications and Future Research

Bacterial antifungal activity observed under a narrow spectrum of laboratory conditions could be lost on exposure to variable field environments. Using antifungal bacteria with inconsistent activity in bioaugmentation efforts could cost managers time and resources, and could create the illusion that bioaugmentation is less effective than if more appropriate isolates were used. Even closely related bacteria may respond differently to environmental variations, as did the Pseudomonads in Figure 3. One produced strongly antifungal metabolites across the entire 8–33°C range, whereas at 8°C others produced metabolites with no antifungal activity.

Only a few studies have characterized the metabolic products of amphibian symbionts [50]–[52]. Based on our observations of varied responses of *Bd* to CFSs from different bacteria, and the phylogenetic range represented in addition to the many Pseudomonads found, it is likely that a variety of different antifungal compounds were produced by the bacteria we tested. As mentioned above, future workers should seek to identify these compounds, so that it is possible to measure their concentrations on amphibian skin.

Our study focused on environmental context-dependent changes in the bacterial production of anti-*Bd* metabolites, but not on possible context-dependent changes in the fungus itself, or in the direct interaction between bacteria and *Bd* on the skins of live frogs. This study constitutes a step towards understanding environmentally induced variation in the amphibian-*Bd*-bacteria symbiosis, but no study has yet simultaneously assessed the responses of both *Bd* and bacteria to varying temperatures. Additionally, no mesocosm or field study of bioaugmentation in a natural environment has been published to date, and a host of questions remain surrounding the best methods of application of beneficial bacteria, non-target effects, and the term of protection afforded. Carrying out *Bd*-bacteria research *in vivo,* and ultimately in natural systems, will be necessary preliminary steps for bioaugmentation application, even if interpretation of specific experimental treatments is complicated. Given the opportunity to apply bioaugmentation for restoration and protection of many amphibian species globally, it is most important to develop effective management protocols for use with robustly-antifungal bacteria commonly found on target species’ skin.

Research on the drivers of other wildlife diseases should also consider the possible effects of environmental context dependence. Some coral disease is exacerbated by warmer environmental temperatures [53]. White-nose syndrome in bats is driven by changes in the temperature of bat hibernacula, which may allow management by artificial temperature regulation in caves [54]. In the case of chytridiomycosis, seasonal and local temperature variation are important, both for their direct effects on the host-pathogen relationship [3], [55] and because they modify relationships within the skin microbe assemblage as described here. The effects of environmental context can thus occur through multiple pathways and can determine the extent to which a disease threatens biodiversity. A thorough understanding of environmental context dependence must therefore be a priority when designing disease management strategies.
